# Draft Genome Sequence of *Anoxybacillus* sp. Strain UARK-01, a New Thermophilic Lignin-Utilizing Bacterium Isolated from Soil in Arkansas, USA

**DOI:** 10.1128/genomeA.00588-17

**Published:** 2017-07-27

**Authors:** Thamir H. Alkahem Albalawi, Douglas D. Rhoads, Ravi D. Barabote

**Affiliations:** a Department of Biological Sciences, University of Arkansas, Fayetteville, Arkansas, USA; b Cell and Molecular Biology Program, University of Arkansas, Fayetteville, Arkansas, USA

## Abstract

The draft genome of *Anoxybacillus* sp. strain UARK-01, a novel lignin-utilizing thermophilic soil bacterium, represents the first sequence of an *Anoxybacillus* isolate from the United States. The genome was sequenced using the Illumina MiSeq platform, *de novo* assembled using SeqMan NGen, and annotated at NCBI. The genome sequence revealed genes for laccase and lignocellulose degradation enzymes.

## GENOME ANNOUNCEMENT

*Anoxybacillus* sp. strain UARK-01 is a thermophilic bacterium that was recently isolated from soil in Arkansas, USA (1). It grows aerobically and optimally at 55°C and pH 8. While *Anoxybacillus* spp. have been isolated mostly from thermal environments, UARK-01 was isolated from soil beneath a grass lawn. Additionally, although *Anoxybacillus* spp. have been isolated and sequenced from around the globe (2–11), strain UARK-01 represents the first genome of an *Anoxybacillus* isolate from the United States. Studies on *Anoxybacillus* isolates are unveiling the potential of this important genus as a new resource for thermostable enzymes (12). Starch, cellulose, xylan, and arabinofuran degradation capabilities have been identified in different *Anoxybacillus* spp.; however, lignin degradation capabilities remain poorly understood in this genus (12). Recently, we showed that strain UARK-01 has the ability to utilize lignin as the sole carbon source and that lignin-grown cultures produce abundant hyperthermostable alkaline laccase activity (1). Here, we describe genome sequencing of the strain UARK-01.

Total genomic DNA was isolated using a Blood & Cell Culture DNA minikit (Qiagen), quantified using a Qubit version 2.0 fluorimeter (Thermo Fisher Scientific), and analyzed using agarose gel electrophoresis. A DNA library was prepared using a Nextera DNA library preparation kit (Illumina), and the genome was shotgun sequenced using paired-end sequencing on a MiSeq Reagent 600-cycle version 3 kit (Illumina). The genome sequence was assembled *de novo* using SeqMan NGen software (DNASTAR) and annotated using the NCBI Prokaryotic Genome Annotation Pipeline (released 2013). Putative genes were analyzed using BLAST (13).

Shotgun genome sequencing yielded a total of 26.7 million pairs of reads, which were assembled into 73 contigs with an average quality score of *Q*35 and average coverage of 311×. The *N*_50_ of the assembly was 120 kb. The largest contig size was 494 kb; 57 contigs were greater than 2.0 kb in length. The total genome size was 3,669,492 bp, and the G+C content was 42.6%. The draft genome encodes 3,672 genes, including 3,377 protein-coding genes, 28 rRNAs, 92 tRNAs, 5 noncoding RNAs (ncRNAs), and 170 pseudogenes. While a single complete rRNA operon encoding 5S, 16S, and 23S rRNA was found, eight additional partial rRNA operons are present in the draft genome. In addition, the genome contains seven CRISPR arrays. Genomes of only three *Anoxybacillus* spp. have been completely assembled, of which two contain plasmids (5–7, 11). None of the plasmid-encoded genes were found in the draft genome of strain UARK-01.

Genes relevant to lignocellulose degradation and metabolism were identified in the genome. Most significantly, genes encoding a laccase enzyme (accession no. KY679089), a putative beta-xylosidase, a xylose isomerase, and two xylose ABC transporters were identified, while no xylanase genes were found. The genome encodes a predicted glycoside hydrolase (GH) belonging to the COG2152 family, which is known to include beta-fructosidases, alpha-l-arabinases, and beta-xylosidases (14). No GH genes related to cellulose degradation were found. These data are consistent with the growth capabilities of strain UARK-01 (1). This genome sequence will facilitate future investigations of lignin metabolism in this bacterium.

### Accession number(s).

This whole-genome shotgun project has been deposited in DDBJ/ENA/GenBank under the accession number NASY00000000. The version described in this paper is the first version, NASY01000000.
